# Hearing Loss: Reestablish the Neural Plasticity in Regenerated Spiral Ganglion Neurons and Sensory Hair Cells

**DOI:** 10.1155/2017/1807581

**Published:** 2017-02-20

**Authors:** Renjie Chai, Geng-Lin Li, Jian Wang, Jing Zou

**Affiliations:** ^1^MOE Key Laboratory of Developmental Genes and Human Disease, Institute of Life Sciences, Southeast University, Nanjing 210096, China; ^2^Co-Innovation Center of Neuroregeneration, Nantong University, Nantong 226001, China; ^3^Biology Department, University of Massachusetts Amherst, Amherst, MA 01003, USA; ^4^School of Human Communication Disorders, Dalhousie University, 1256 Barrington Street, Halifax, NS, Canada B3J1Y6; ^5^Department of Otolaryngology-Head and Neck Surgery, Changhai Hospital, Second Military Medical University, Shanghai 200433, China; ^6^Hearing and Balance Research Unit, School of Medicine, University of Tampere, Lääkärinkatu 1, Room E222, 33520 Tampere, Finland

Hearing loss is considered as the most common sensory disorder in human population that occurs at all ages world-widely and sensorineural hearing loss (SNHL) is the most common type of hearing loss. Various insults could induce SNHL, including acoustic trauma, ear and brain tumors, aging, noise exposure, or ototoxic medications or chemicals. SNHL is caused by irreversible loss of sensory hair cells and/or degeneration of spiral ganglion neurons. SNHL is not yet curable due to the lack of automatic regeneration of hair cells and spiral ganglion neurons in the cochlea. In recent years, exciting animal studies on signaling pathway manipulation, gene therapy, and stem cell transplantation as well as pharmaceutical agents have demonstrated that hair cells and spiral ganglion neurons could be triggered to regenerate, suggesting that hearing loss might be curable eventually in the future. Neural plasticity is the key feature for hair cells and spiral ganglion neurons, and it is especially important for the newly regenerated hair cells and spiral ganglion neurons to be functionally integrated into auditory pathways. In this special issue on neural plasticity of hair cells and spiral ganglion neurons, we are pleased to present a series of articles that represent the latest advances in hair cell development, protection and regeneration, spiral ganglion neuron development and protection, and inherited hearing loss.

*Hair Cell Development.* J. Hang et al. (“Synchronized Progression of Prestin Expression and Auditory Brainstem Response during Postnatal Development in Rats”) report that the onset time of hearing may require the expression of prestin and is determined by the functional maturation of outer hair cells. H. Nie et al. (“Plasma Membrane Targeting of Protocadherin 15 Is Regulated by the Golgi-Associated Chaperone Protein PIST”) report that PIST regulates the intracellular trafficking and membrane targeting of the tip-link proteins CDH23 and PCDH15 in hair cells. 

*Hair Cell Damage and Hair Cell Protection*. X. Liu et al. (“Analysis of the Damage Mechanism Related to CO_2_ Laser Cochleostomy on Guinea Pig Cochlea”) report that enhanced cell-cell adhesion and activation of *β*-catenin-related canonical Wnt signaling pathway may play a role in the protection of the cochlear from further damage. M. Fu et al. (“The Effects of Urethane on Rat Outer Hair Cells”) report that urethane anesthesia is expected to decrease the responses of outer hair cells, whereas the frequency selectivity of the cochlea remains unchanged. X. Fu et al. (“Loss of Myh14 Increases Susceptibility to Noise-Induced Hearing loss in CBA/CaJ Mice”) report that Myh14 may play a beneficial role in the protection of the cochlea after acoustic overstimulation in CBA/CaJ mice. L. Shi et al. (“Cochlear Synaptopathy and Noise-Induced Hidden Hearing Loss”) provide a brief review to address several critical issues related to NIHHL: mechanisms of noise-induced synaptic damage, reversibility of synaptic damage, functional deficits in NIHHL animal models, evidence of NIHHL in human subjects, and peripheral and central contributions of NIHHL. 

*Hair Cell Regeneration.* Y. Shu et al. (“Adenovirus Vectors Target Several Cell Subtypes of Mammalian Inner Ear* In Vivo*”) report that adenovirus vectors are capable of efficiently and specifically transfecting different cell types in the mammalian cochlea and therefore provide useful tools to study inner ear gene functions and evaluate gene therapies for treating hearing loss and vestibular dysfunction. X. Lu et al. (“Mammalian Cochlear Hair Cell Regeneration and Ribbon Synapse Reformation”) review recent research progress in hair cell regeneration, synaptic plasticity, and reinnervation of new regenerated hair cells in the mammalian cochlea. C. Wang et al. (“Evaluation of the Hair Cell Regeneration in Zebrafish Larvae by Measuring and Quantifying the Startle Responses”) report the capability of a behavioral assay in noninvasively evaluating hair cell functions of fish larvae and its potential as a high-throughput screening tool for auditory-related gene and drug discovery. 

*Spiral Ganglion Neuron Development and Protection*. P. Chen et al. (“NLRP3 Is Expressed in the Spiral Ganglion Neurons and Associated with Both Syndromic and Nonsyndromic Sensorineural Deafness”) report that NLRP3 may have specific functions in spiral ganglion neurons that are altered in both syndromic and nonsyndromic sensorineural deafness. X. Bai et al. (“Protective Effect of Edaravone on Glutamate-Induced Neurotoxicity in Spiral Ganglion Neurons”) investigated the toxicity of glutamate in spiral ganglion neurons and they found that the protection of edaravone is related to the PI3K pathway and Bcl-2 protein family. 

*Inherited Hearing Loss*. X. Gu et al. (“Massively Parallel Sequencing of a Chinese Family with DFNA9 Identified a Novel Missense Mutation in the LCCL Domain of COCH”) identified a missense mutation in the LCCL domain of COCH that is absent in 100 normal hearing controls and cosegregated with impaired hearing. J. Chen et al. (“Identification of a Novel ENU-Induced Mutation in Mouse* Tbx1* Linked to Human DiGeorge Syndrome”) confirm the pathogenic basis of* Tbx1* in DGS, point out the crucial role of DNA binding activity of* Tbx1* for the ear function, and provide additional animal model for studying mechanisms underlying the DGS disease. Y. Guo et al. (“The Relative Weight of Temporal Envelope Cues in Different Frequency Regions for Mandarin Sentence Recognition”) report that, for Mandarin Chinese, a tonal language, the temporal E cues of Frequency Region 1 (80–502 Hz) and Region 3 (1,022–1,913 Hz) contribute more to the intelligence of sentence recognition than other regions, particularly the region of 80–502 Hz, which contains fundamental frequency (*F*0) information. L. He et al. (“Mutation in the Hair Cell Specific Gene* POU4F3* Is a Common Cause for Autosomal Dominant Nonsyndromic Hearing Loss in Chinese Hans”) report that mutations in* POU4F3* are a relatively common cause (3/16) for ADNSHL in Chinese Hans, which should be routinely screened in such cases during genetic testing. C. Zhang et al. (“A Novel Nonsense Mutation of* POU4F3* Gene Causes Autosomal Dominant Hearing Loss”) report the first nonsense mutation of* POU4F3* associated with progressive hearing loss and explored the possible underlying mechanism.

The articles in this special issue provide valuable insights into development, protection, and regeneration of hair cells and spiral ganglion neurons. By highlighting findings in these articles, we hope this special issue will provide not only new perspectives for future directions in hearing research but also potential therapeutic strategies for treating hearing loss.

*Renjie Chai* *Geng-Lin Li* *Jian Wang* *Jing Zou*

